# The actin binding proteins cortactin and HS1 are dispensable for platelet actin nodule and megakaryocyte podosome formation

**DOI:** 10.1080/09537104.2016.1235688

**Published:** 2016-10-25

**Authors:** Steven G. Thomas, Natalie S. Poulter, Danai Bem, Brenda Finney, Laura M. Machesky, Stephen P. Watson

**Affiliations:** ^a^ Institute of Cardiovascular Sciences, Institute for Biomedical Research, The Medical School, University of Birmingham, Edgbaston, Birmingham, UK; ^b^ Cancer Research UK Beatson Institute, College of Medical. Veterinary and Life Sciences, University of Glasgow, Glasgow, UK

**Keywords:** Actin nodules, Cortactin, HS1, platelets, podosomes

## Abstract

A dynamic, properly organised actin cytoskeleton is critical for the production and haemostatic function of platelets. The Wiskott Aldrich Syndrome protein (WASp) and Actin-Related Proteins 2 & 3 Complex (Arp2/3 complex) are critical mediators of actin polymerisation and organisation in many cell types. In platelets and megakaryocytes, these proteins have been shown to be important for proper platelet production and function. The cortactin family of proteins (Cttn & HS1) are known to regulate WASp-Arp2/3-mediated actin polymerisation in other cell types and so here we address the role of these proteins in platelets using knockout mouse models.

We generated mice lacking Cttn and HS1 in the megakaryocyte/platelet lineage. These mice had normal platelet production, with platelet number, size and surface receptor profile comparable to controls. Platelet function was also unaffected by loss of Cttn/HS1 with no differences observed in a range of platelet function assays including aggregation, secretion, spreading, clot retraction or tyrosine phosphorylation. No effect on tail bleeding time or in thrombosis models was observed. In addition, platelet actin nodules, and megakaryocyte podosomes, actin-based structures known to be dependent on WASp and the Arp2/3 complex, formed normally. We conclude that despite the importance of WASp and the Arp2/3 complex in regulating F-actin dynamics in many cells types, the role of cortactin in their regulation appears to be fulfilled by other proteins in platelets.

## Introduction

The actin cytoskeleton plays a critical role in both the production of platelets from megakaryocytes [1] and in the proper functioning of platelets [2]. Several proteins, which are either part of the cytoskeleton or regulate its organisation (including mDia1, WASp, Myosin, profilin and ADF/Cofilin), have been shown to be important for producing sufficient platelets of the correct size [3–9]. Similarly, many cytoskeletal proteins have been shown to be required for proper platelet function including Filamin A, WASp, myosin, and α-actinin [5,10–12]. Two actin-related structures which have been identified in platelets and megakaryocytes are the actin nodule and the podosome, respectively [7,13]. Recent studies have shown that podosomes, and the subsequent degradation of extracellular matrix, are required for proplatelet protrusion through the basement membrane of the blood vessel during the later stages of platelet formation [14], and that actin nodules are platelet podosome-like structures which contribute to platelet aggregate stability under flow [11]. Both of these structures are dependent on WASp-Arp2/3-mediated actin polymerisation for proper formation [7,11,13,14]. Furthermore, the Arp2/3 complex is regulated by a number of different proteins including the cortactin (Cttn) family of proteins (Cttn & haematopoietic lineage cell-specific protein 1 (HS1)) [15].

Cttn is an F-actin binding protein which can interact with the actin nucleation promoting factor, the Arp2/3 complex and stabilise dynamic branched actin networks. In addition, it can act as a scaffolding protein due to its ability to interact with a wide range of molecules (Reviewed in [15–17]). It is a target for both kinases and acetylases, post-translational modifications which regulate its F-actin binding activity and subsequent role in dynamic actin processes. Cttn is ubiquitously expressed, however, in haematopoietic cells its homologue HS1 is the major form. Osteoclasts and megakaryocytes/platelets are unusual in that both forms are expressed [18] and their expression has been shown to increase during megakaryocyte maturation [19]. In addition, Cttn has been reported to be robustly phosphorylated in human platelets following activation [20–23]. Studies where Cttn expression has been knocked out in mouse embryonic fibroblasts have produced conflicting results showing either no effect on actin dynamic and cell motility or a reduction in these processes [24,25]. Several studies have shown that Cttn can regulate the formation of podosomes and invadopodia (reviewed in [17]) and that loss of Cttn reduces the invasiveness of cancer cells through reduction in invadopodia formation and ECM degradation [26]. We have previously shown that platelets deficient in HS1 are indistinguishable from controls [27], although Kahner et al. [28] showed a small reduction in platelet function. Furthermore, recent evidence suggests that Cttn affects proplatelet production in human megakaryocytes, but in mice, megakaryocyte development and proplatelet formation is unaffected by loss of Cttn (unpublished data). Thus, Cttn may have a minor role in megakaryocyte and platelet dynamics, but this is likely to be redundant with other proteins for most purposes.

To address whether Cttn and the related protein HS1 showed redundancy, we generated a megakaryocyte/platelet specific gene knockout mouse (Cttn KO) which we crossed with the HS1 knockout mouse to generate Cttn/HS1 double knockouts (DKO). Here, we present evidence that loss of both Cttn and HS1 shows no detectable phenotype in platelets, suggesting further redundancy in the system and calling into question reports on the essential nature of the Cttn-related proteins in cell dynamics.

## Methods

### Generation of a Cttn/HS1 double knockout mouse

HS1 knockout mice were generated and genotyped as described previously [27,29]. Conditional Cttn knockout mice were generated by Taconic (Koeln, Germany) (Supplementary Figure 1A, B, C). Conditional Cttn mice were crossed with the PF4-Cre recombinase (PF4-Cre) expressing mouse line [30] and the HS1 knockout mice to generate either platelet-specific Cttn knockout mice (Cttn KO) or platelet-specific Cttn and constitutive HS1 knockout mice (DKO). Wildtype mice (WT) were either conditional Cttn mice which expressed no PF4-Cre recombinase, or PF4-Cre positive mice which lacked the floxed Cttn allele. Genotyping of mice was carried out by PCR on genomic DNA extracted from ear clippings taken at three weeks after birth. Primers PF4Cre-R (5′-TGCACAGTCAGCAGGTT-3′) and Pf4Cre-F (5′-CCCATACAGCACACCTTTTG-3′) were used to identify mice containing the PF4-Cre recombinase (~400 bp fragment). For Cttn, WT and floxed alleles were identified using forward and reverse primers (5′-TCATCAAGATCGGTGGTTCC-3′ & 5′-CAGTGATGGACTTAGAAGCTGG-3′, respectively) to generate 284 bp WT or 441 bp floxed PCR fragments (Supplementary Figure 1D). Western blotting confirmed loss of protein expression in mouse platelets (Supplementary Figure 1E). The genotypes used in this study are indicated in Supplemental Figure 1F. All animals were maintained using housing and husbandry in accordance with local and national legal regulations.

**Figure 1.**
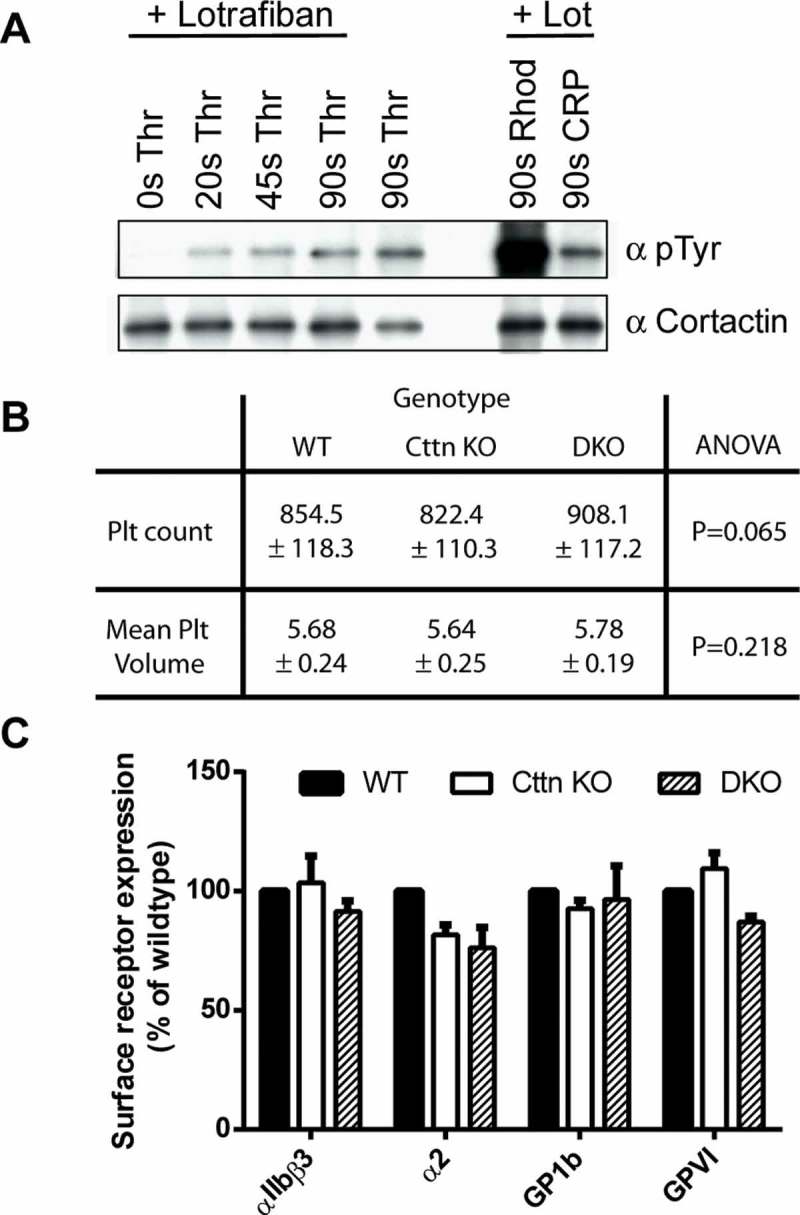
Characterisation of platelets from Cttn KO and DKO mice. (A) Western blots of platelet lysates confirming phosphorylation of Cttn downstream of G-protein coupled and tyrosine kinase linked receptors. Cortactin was immuno-precipitated from stimulated mouse platelet lysates and western blotted with anti-phosphotyrosine (4G10) and anti-cortactin (4F11) antibodies. (B) Whole blood platelet counts and mean platelet volumes were measured from WT, Cttn and DKO mice. No significant difference was observed between the genotypes in either platelet number or volume. Data is mean ± SD (WT, *n* = 37; Cttn KO, *n* = 24; DKO, *n* = 18). (C) Platelet surface receptor levels were analysed by flow cytometry. No significant differences were observed between either genotype and WT controls. Data are % of WT control (Mean ± SEM; *n* = 3) and values were corrected for IgG background staining.

### Preparation of mouse platelets

Blood was drawn from CO_2_ terminally-narcosed mice under anaesthesia from the vena cava and taken into ACD at a ratio of 1:10 for washed platelets or, for studies performed in platelet-rich plasma (PRP), into sodium citrate. Platelet numbers in whole blood were determined using an ABX Micros 60 (ABX Diagnostics, Montpelier, France). PRP and washed platelets were prepared as previously described [31].

### DIC and fluorescence microscopy of spread platelets

Coverslips were incubated with a suspension of fibrinogen (100 μg mL^−1^) or collagen-related peptide (CRP, 100 μg mL^−1^) overnight at 4°C. Surfaces were washed and then blocked with denatured BSA (5 mg mL^−1^) for 1 h at room temperature followed by subsequent washing with PBS before use in spreading assays. Platelets (2 x 10^7^ mL^−1^) were allowed to spread on immobilized proteins for 45 min at 37°C. Surfaces were then washed with PBS to remove non-adherent cells before fixation with 10% formalin (4% paraformaldehyde), for 10 min at room temperature. Platelet morphology was imaged as previously described [31]. The platelet surface area of spread platelets was computed using Fiji software package (http://fiji.sc/#). For actin staining: fixed, spread platelets were permeabilised with 0.1% Triton X-100 in PBS for 5 min, washed 3x in PBS and then stained with 488-phalloidin (1 in 500) for 60 mins before imaging.

### Platelet aggregation studies

Platelet aggregation was monitored using 300 μL of 2 x 10^8^ mL^–1^ of washed platelets. Stimulation of platelets was performed in a Chrono-Log aggregometer (Chrono-Log, Havertown, PA, USA) with continuous stirring at 1200 rpm at 37 °C as previously described [32]. ATP secretion was also determined during aggregation using Chronolume reagent.

### Flow cytometry studies

Surface receptor levels on washed platelets were determined using anti-mouse antibodies (Emfret) against αIIbβ3 (clone LeoF2), GP1b (clone Xia.H10), α2 (clone SAMC1) and GPVI (clone JAQ1). P-Selectin expression, phosphatidylserine exposure and fibrinogen binding were determined using anti-P-Seletin (Emfret, clone Wug.Eg), Annexin-V-PE (BD Biosciences) and 488-Fibrinogen (Invitrogen), respectively. Samples were analysed using an Accuri C6 flow cytometer (BD Biosciences).

### Clot retraction assays

Whole murine blood was anti-coagulated with sodium citrate and PRP prepared as above. The platelet count was adjusted to 3 × 10^8^ /ml with HEPES-Tyrodes containing CaCl_2_ (2 mM) and fibrinogen (2 mg/ml). 400 μl of this mix was placed into an aggregometer tube and incubated at 37°C for 5 min. 2 μl of mouse erythrocytes were added for colour contrast. Thrombin (10 U/ml) was added and mixed with a paper-clip and clot retraction was allowed to proceed at 37°C for 1 hour with the paper-clip present. At appropriate time points, the clot was pulled out with the paper-clip and the remaining serum volume measured. These experiments were performed blind.

### Tail bleed assays

Experiments were conducted on 20–35 g male and female WT (*n* = 18), Cttn KO (*n* = 12) and DKO (*n* = 7) mice. Mice were anaesthetized with isofluorane via a face mask throughout the experiment and subsequently injected with the analgesic buprenorphine (ip). The terminal 3 mm of tail was removed using a sharp razor blade and blood was collected. Mice were allowed to bleed until they lost either 15% blood volume or for 20 min. Data were presented as the weight of blood lost (mg).

### In vitro flow studies

For *in vitro* flow studies, mouse blood was prepared and treated as described by Calaminus *et al*. [31]. Platelet adhesion results are expressed as the percentage of surface area covered by platelets.

### Laser injury thrombosis model

The ability of Cttn KO mice to undergo normal thrombosis was tested using the laser injury model as previously described [33].

### Megakaryocyte podosome formation

Bone marrow megakaryocytes were isolated and allowed to spread on fibrinogen coated coverslips for 3 hrs as previously described [34], prior to staining with 488-phalloidin and anti-vinculin.

### Data analysis

Results are shown as mean ± SEM from at least three experiments unless otherwise stated. Statistical comparisons were made using ANOVA or Student’s test as appropriate using Graphpad Prism 6.

## Results and discussion

### Generation of knockout mice

Cttn has been reported to be robustly phosphorylated in human platelets following activation [20–23] and we confirmed that Cttn was also phosphorylated in mouse platelets downstream of both tyrosine kinase linked and G protein-coupled receptors (Figure 1A). We had previously demonstrated that genetic knockout of HS1 in mouse platelets has no effect on platelet function [27]. A similar study by Kahner et al. [28], on the same mouse model, identified a mild bleeding defect in the HS1 knockout mice. We hypothesised that the absence of a significant phenotype in these mice may be due to the expression of the second family member, Cttn in megakaryocytes and platelets [19,35]. As evidence suggested that Cttn was important at a very early stage of oocyte development [36], we made a conditional flox mouse. This mouse expressed a Cttn allele where exon 5 was flanked by LoxP sites using homologous recombination (Supplementary Figure 1A & B). Removal of exon 5 from Cttn generates a premature stop codon in exon 7 which results in a truncated transcript which is predicted to undergo nonsense mediated RNA decay (Supplementary Figure 1C). These mice were crossed with mice expressing the megakaryocyte-specific PF4-Cre recombinase transgene [30] to generate platelet- and megakaryocyte-specific cortactin knockout mice (Cttn KO) and the HS1 knockout mice to generate double Cttn/HS1 knockout mice (DKO). Mice were genotyped using PCR (Supplementary Figure 1D) with the genotypes of the mice used in this study shown in Supplementary Figure 1E).

### Characterisation of platelets from knockout mice

Both Cttn KO and DKO mice displayed no overt phenotype and observation of the mice revealed no obvious defects in development. Furthermore, knockout mice were visually undistinguishable from WT or heterozygous mice. To establish that gene disruption resulted in a loss of expression of protein, western blots were performed confirming that expression of Cttn and/or HS1 proteins was lost in platelets (Supplementary Figure 1E). Whole blood platelet analysis was performed and no difference was observed in either platelet number or volume (Figure 1B) between WT, Cttn KO and DKO mice, indicating that the steady-state production of platelets is not affected by the loss of these proteins. Other haematological parameters, including red blood cell count, haematocrit, mean corpuscular volume and white blood cell count, were also within normal ranges (data not shown). Furthermore, both Cttn KO and DKO platelets had normal levels of the major platelet surface receptors (αIIbβ3, α2β1, GP1b & GPVI, Figure 1C). These data show that platelet production is normal in both Cttn KO and DKO mice.

### Platelet spreading and F-actin organisation are unaffected by loss of Cttn and HS1

**Figure 2.**
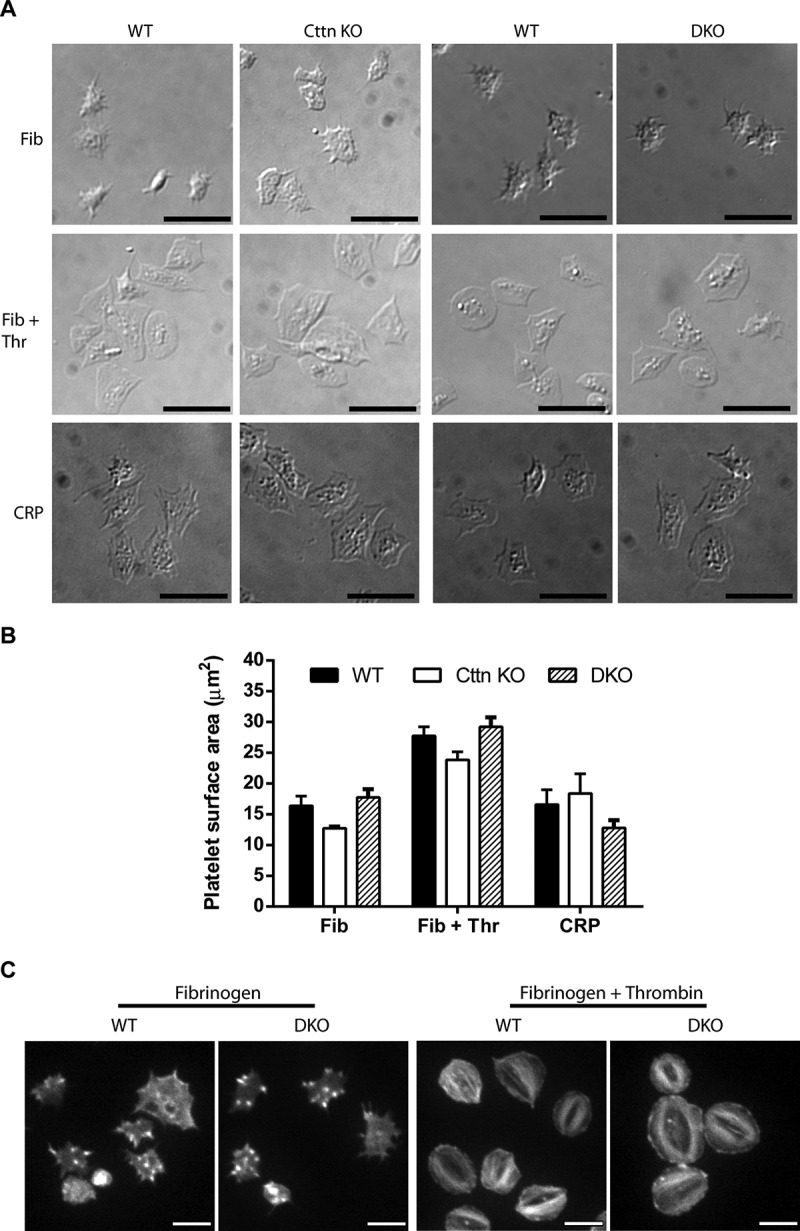
Loss of Cttn and HS1 does not affect platelet spreading or F-actin organisation. (A) The loss of Cttn only or Cttn and HS1 from platelets did not affect their ability to adhere and spread on fibrinogen (±0.1 U/ml thrombin) or collagen related peptide (CRP) coated coverslips. (B) Quantitation of the surface area of spread platelets from either Cttn KO or DKO platelets showed no significant differences in spreading. (C) Staining of spread platelets for F-actin with fluorescent phalloidin showed normal actin organisation with filopodia, actin nodules and platelet stress fibres being observed in both WT and DKO platelets. Scale bars in (A) = 10 µm and in (C) = 5 µm.

Cttn and HS1 are known to interact with F-actin and a number of actin-binding proteins (including Arp2/3 complex, WASp, WIP, dynamin) as well as with other signalling proteins (e.g. Src family kinase, PTP1B & PAK) [15,16]. They are believed to be key in organising the actin cytoskeleton and the formation of actin-rich structures including lamellipodia, actin nodules and podosomes. Therefore, we investigated the effect of loss of Cttn and HS1 on platelet spreading, a process dependent on F-actin dynamics and organisation. No significant difference was observed for either Cttn KO or DKO platelets compared to WT controls when spread on fibrinogen (± thrombin) or collagen-related peptide (CRP) (Figure 2A & B). Furthermore, Cttn KO and DKO platelets formed normal filopodia and actin nodules during early spreading on fibrinogen and went onto form both lamellipodia and stress fibres when fully spread (Figure 2C) with normal Arp2/3 localisation (Supplementary Figure 4A & B). These data indicate that neither protein is essential for normal platelet spreading and highlights the redundancy present within platelets regarding actin organisation. Furthermore, it is interesting that while both the Arp2/3 complex and WASP are required for actin nodule formation [11], Cttn is not.

**Figure 3.**
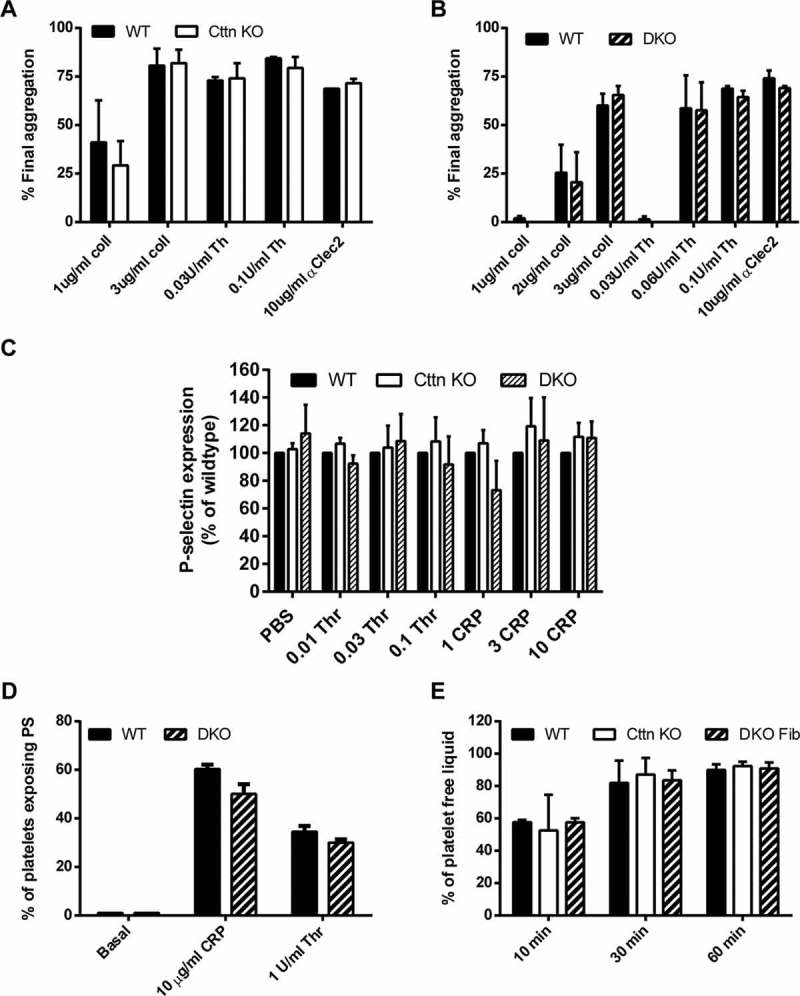
Loss of Cttn and HS1 does not affect platelet function. The loss of Cttn alone or both Cttn and HS1 had no effect on the aggregation (A & B) or α-granule secretion (C) of platelets to G-protein coupled or tyrosine kinase linked receptor agonists. Aggregation data are expressed as % final aggregation 6 mins after agonist addition. Alpha granule secretion data are % of WT values measured by flow cytometry 2 mins after addition of agonist. (D) The loss of Cttn and HS1 had no effect on pro-coagulant surface generation as phosphatidylserine exposure was not affected by loss of both cortactin and HS1. (E) Clot retraction in PRP following stimulation by thrombin was unaffected by loss of Cttn or Cttn and HS1. All data are presented as mean ± SEM, *n* = 3.

**Figure 4.**
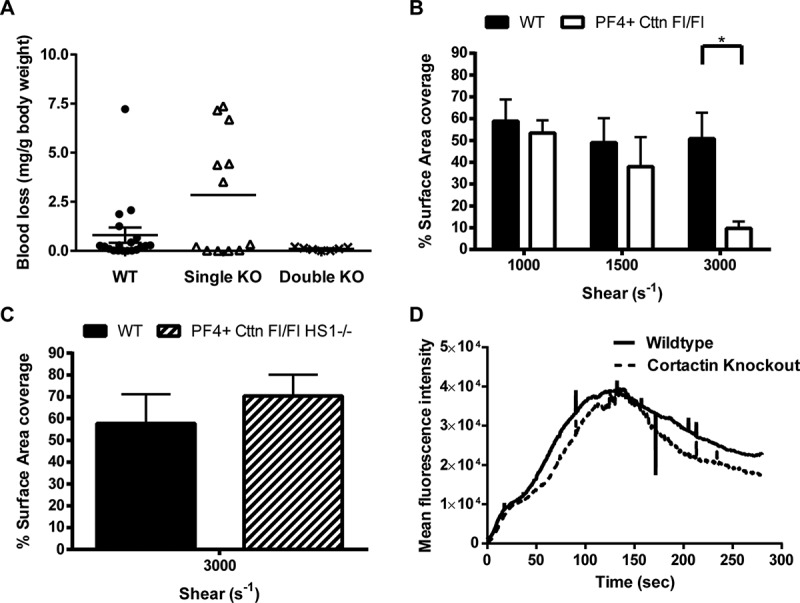
In vivo thrombosis assays and in vitro flow studies. (A) An increase in tail bleeding (mg blood loss/g body weight) was observed in Cttn KO mice following removal of the terminal 3 mm of the tail. However, this increase was not significant and was not observed in DKO mice. Symbols (● = WT, Δ = Cttn KO, × = DKO) represent individual data points, horizontal bars the mean and vertical bars the SEM (*n* = 18 for WT, 12 for Cttn KOs and 7 for DKOs). (B) *In vitro* flow assays performed over collagen showed no significant decrease in aggregate formation at shear rates of either 1000 s^−1^ or 1500 s^−1^ for either Cttn KO or DKO mice. (C & D) In vivo thrombosis, as determined by the cremaster laser injury model, showed no effect of loss of Cttn on either thrombus size (C) or time to peak intensity (D).

### Loss of Cttn and HS1 does not affect platelet function

To fully assess the effect of genetic deletion of Cttn and HS1 on platelet function, washed platelets were tested for a number of functional responses. First, the ability of platelets to aggregate in response to either collagen or thrombin was monitored at both low and high doses of agonist. For both agonists and at all doses, no significant difference was observed between WT, Cttn KO or DKO platelets (Figure 3A & B). The absence of an aggregation defect was confirmed by measuring αIIbβ3 integrin activation via fluorescent fibrinogen binding. Data indicate that there was no difference in integrin activation between WT, Cttn KO or DKO platelets (Supplementary Figure 2C). Secretion of platelet granule contents is critical for proper platelet function and the actin cytoskeleton is important for this process [37]. To establish if there is any deficiency in granule secretion, we monitored release of dense granules and α-granules during platelet activation. No significant difference was observed in the secretion of either dense granule contents (measured by observing ATP secretion during platelet activation - Supplementary Figure 2A & B) or in α-granule contents (measured by observing P-selectin expression on the platelet surface – Figure 2C) following platelet activation downstream of tyrosine-kinase or G protein coupled receptor agonists. Finally, signal transduction in the platelets was measured by observing phosphotyrosine signalling downstream of collagen activation. No difference observed between DKO and WT platelets (Supplementary Figure 2D).

The ability of platelets to link to the coagulation cascade and provide a pro-thrombotic surface was also investigated. The expression of phosphatidylserine on the platelet surface was unaffected by the loss of Cttn or both Cttn and HS1 (Figure 3D), and the ability of mutant platelets to drive clot retraction was also not significantly different from controls (Figure 3E). All together, these data indicate that although Cttn is an important regulator of F-actin network dynamics and scaffolding in many cell types in vitro, it does not appear to be required for proper platelet function.

### Physiological and in vivo platelet function

To establish if mice lacking Cttn displayed impaired haemostasis, tail bleeding time measurements were performed. Single Cttn KO mice displayed a slightly reduced haemostatic capacity as the amount of blood lost was increased when compared to both WT and DKO mice (Figure 4A). However, this difference was not significant. Furthermore, in flow aggregation experiments, no difference was observed in surface area coverage of platelet aggregates for WT and Cttn or DKO mice at either 1000 s^−1^ or 1500 s^−1^ (Figure 4B). In addition, no difference in the dynamics of thrombus formation or in time to peak intensity (Figure 4C & D) was observed in the laser injury thrombosis model. Taken together, this data suggests that platelet Cttn and HS1 are not required for normal haemostasis.

### Podosome formation in bone marrow megakaryocytes

Cttn is implicated in the formation of podosomes and invadopodia, actin-rich structures involved in extracellular-matrix interactions [38] and we have previously shown that podosomes are required for proper proplatelet protrusion across the basement membrane [14]. Bone marrow derived megakaryocytes from WT, Cttn KO and DKO mice spread on fibrinogen all produced podosomes with the characteristic vinculin ring staining pattern (Supplementary Figure 3) and normal Arp2/3 localisation (Supplementary Figure 4C & D). Megakaryocytes from DKO mice develop normally as assessed by total MK number, number of CFU-MKs, DNA ploidy level and are able to undergo normal proplatelet formation and platelet release (data not shown). Furthermore, in these mice, steady-state platelet number and size was normal (Figure 1B) and platelet recovery following immune-induced thrombocytopenia was also normal (data not shown). Taken together, these data indicate that loss of Cttn and HS1 following genetic knock out does not affect megakaryocyte development or platelet production.

In conclusion, while Cttn is well described in the literature as a regulator of F-actin network dynamics in several cell types and specifically to play a role in invadopodia/podosome formation [39, 40, 41], the data presented here demonstrate that Cttn and HS1 are redundant for function in mouse megakaryocytes and platelets. It is possible that some of the redundancy in genetic knockouts such as HS1 and Cttn reflects compensation following long term loss of these proteins. Most cell biology studies of Cttn in regulation of the actin cytoskeleton involve relatively short term siRNA knockdowns, while we have used long term genetic knockout. It is increasingly becoming apparent that cells and perhaps also tissues and organisms often can compensate for loss of gene function by modification of other pathways [42]. Timescale must also be considered when comparing inhibitors and genetic knockouts due to potential compensation mechanisms. Thus, Cttn and HS1 may play a role in platelet and megakaryocyte function, but this may be compensated for when they are deleted in the long term.

Not all actin organisers are redundant, however, as loss of WASp gives a mild but measurable phenotype [11]. Shorter term, inhibition of platelet Arp2/3 complex completely blocks platelet spreading and actin nodule formation [11], but this has not been tested by genetic knockout. It would be interesting to know whether WASp, Cttn, HS1 triple null platelets had a more severe defect than WASp nulls, but this experiment would be technically difficult to achieve. Furthermore, it would be interesting to know if loss of Arp2/3 complex could be genetically compensated for, as loss of Arp2/3 in fibroblasts causes major phenotypes [42,43,44].

## Supplementary Material

Thomas et al Supplemental Figures
