# Supplementary material for: The actin binding proteins cortactin and HS1 are dispensable for platelet actin nodule and megakaryocyte podosome formation
Source: Platelets. 2016 Oct 25;28(4):372–9. doi: 10.1080/09537104.2016.1235688 (PMC5274539; doi:10.1080/09537104.2016.1235688)
Supplement: Thomas et al Supplemental Figures [file iplt_a_1235688_sm0637.zip › SuppFig-Captions.docx]

**Supplementary Fig 1. Generation of Cttn knockout mice.**

(A) Schematic representation of mouse Cttn and HS1 proteins. (N – terminal acidic domain, R1, R2, etc – Cortactin repeats, CC – coiled coil helical domain, PRD – proline rich domain, SH3 – C-terminal Src homology domain. Numbers indicate amino acid number. (B) Targeting strategy of mouse Cttn gene to generate conditional allele. Exon 5 was flanked with LoxP sites allowing excision by Cre recombinase. (C) Deletion of exon 5 of Cttn generates a premature stop codon in exon 7 resulting in a truncated transcript that is predicted to undergo non-sense mediated RNA decay. (D) PCR genotyping of Cttn, HS1 and PF4-Cre recombinase mice. PF4-Cre positive mice gave a ~400kb band. The WT Cttn allele gave rise to a 284bp band whilst the floxed conditional allele gave a 441bp band. For HS1 the WT allele gave a 1.2kb band with the KO allele giving a 1.1Kb band. (E) Western blots confirming loss of expression of Cttn and HS1 proteins in mouse platelets. (4F11 anti-cortactin, Anti-HS1 [39]) (F) Mouse genotypes used in the study.

**Supplementary Fig 2. Platelet function tests**

The loss of (A) Cttn or (B) Cttn and HS1 from mouse platelets had no effect on the secretion of ATP from dense granules. (C) The activation of αIIbβ3, as measured by fluorescent fibrinogen binding, was also unaffected by the loss of either Cttn or Cttn and HS1. Integrin activation is presented as % of WT values measured by flow cytometry 2 mins after addition of agonist and dense granule secretion was determined by measuring ATP release from platelets using Chonolume reagent. Data are presented as mean ±SEM, n = 3. (D) Phosphotyrosine signalling downstream of collagen stimulation was also unaffected by the loss of Cttn and HS1 (4G10; anti-phosphotyrosine).

**Supplementary Fig 3. Loss of Cttn and HS1 has no effect on megakaryocytes and platelet production.**

Bone marrow derived megakaryocytes from Cttn KO (middle column) and DKO (right column) mice were both able to form podosomes when spread on fibrinogen (top row). Close up of regions containing podosomes from all genotypes displayed an actin rich core (second row) and vinculin ring (third row) staining pattern characteristic of megakaryocyte podosomes. The bottom row shows the merged image with examples of podosomes arrowed. Scale bars = 10μm.

**Supplementary Fig 4. Organisation of Arp2/3 complex is normal in platelets and megakaryocytes from DKO mice.**

Platelets spread on fibrinogen (Fib) or fibrinogen ± thrombin (Fib + Thr) from (A) WT or (B) DKO mice show normal localisation of Arp2/3 complex at actin nodules and lamellipodia. Megakaryocytes spread on fibrinogen from (C) WT or (D) DKO mice also show normal Arp2/3 localisation at podosomes and lamellipodia. In all images green = Arp2/3 complex and magenta = F-Actin.
