# Supplementary figures and images for: The actin binding proteins cortactin and HS1 are dispensable for platelet actin nodule and megakaryocyte podosome formation
Source: Platelets. 2016 Oct 25;28(4):372–9. doi: 10.1080/09537104.2016.1235688 (PMC5274539; doi:10.1080/09537104.2016.1235688)

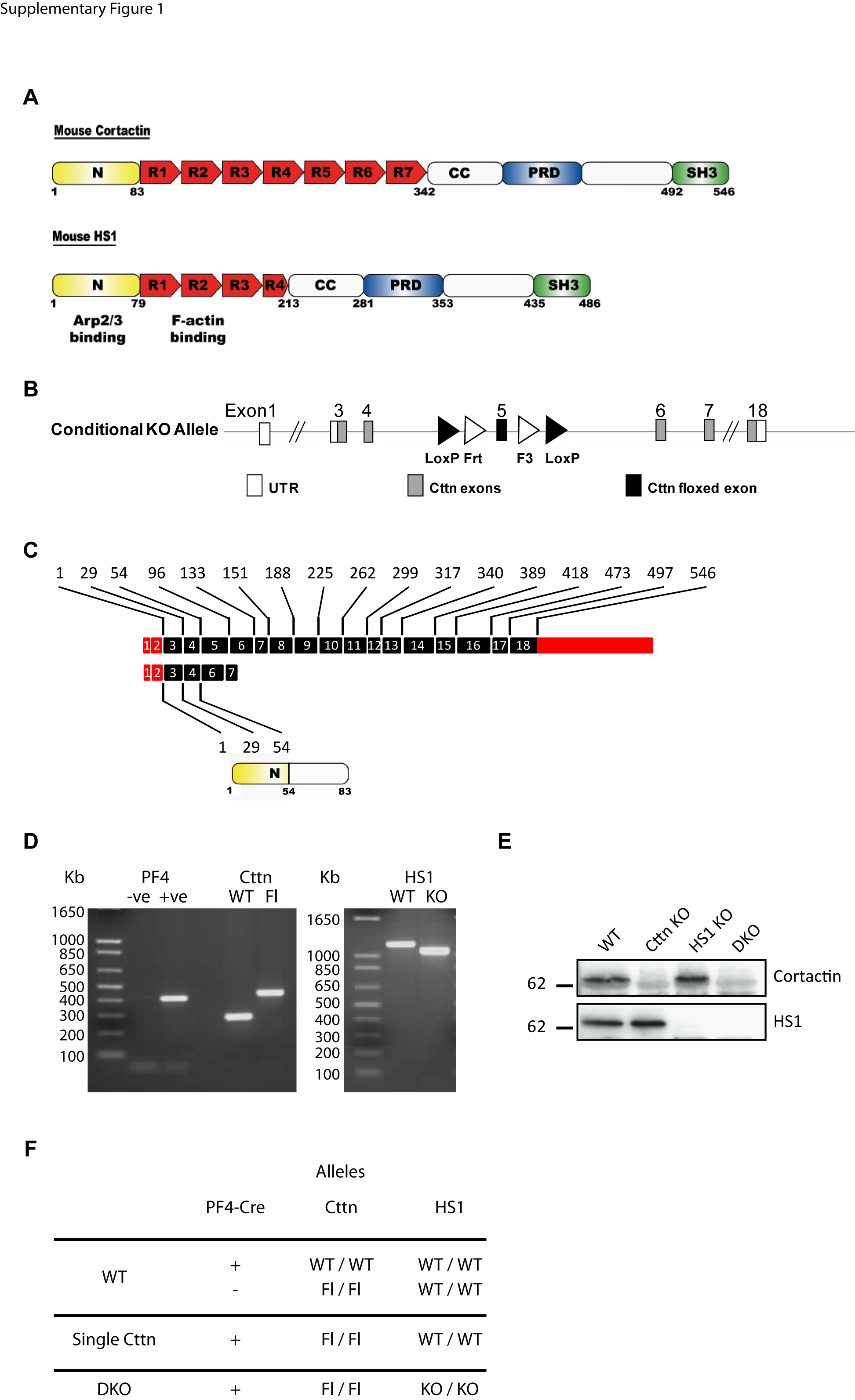

Supplement: Thomas et al Supplemental Figures [file iplt_a_1235688_sm0637.zip › SuppFig1.png]

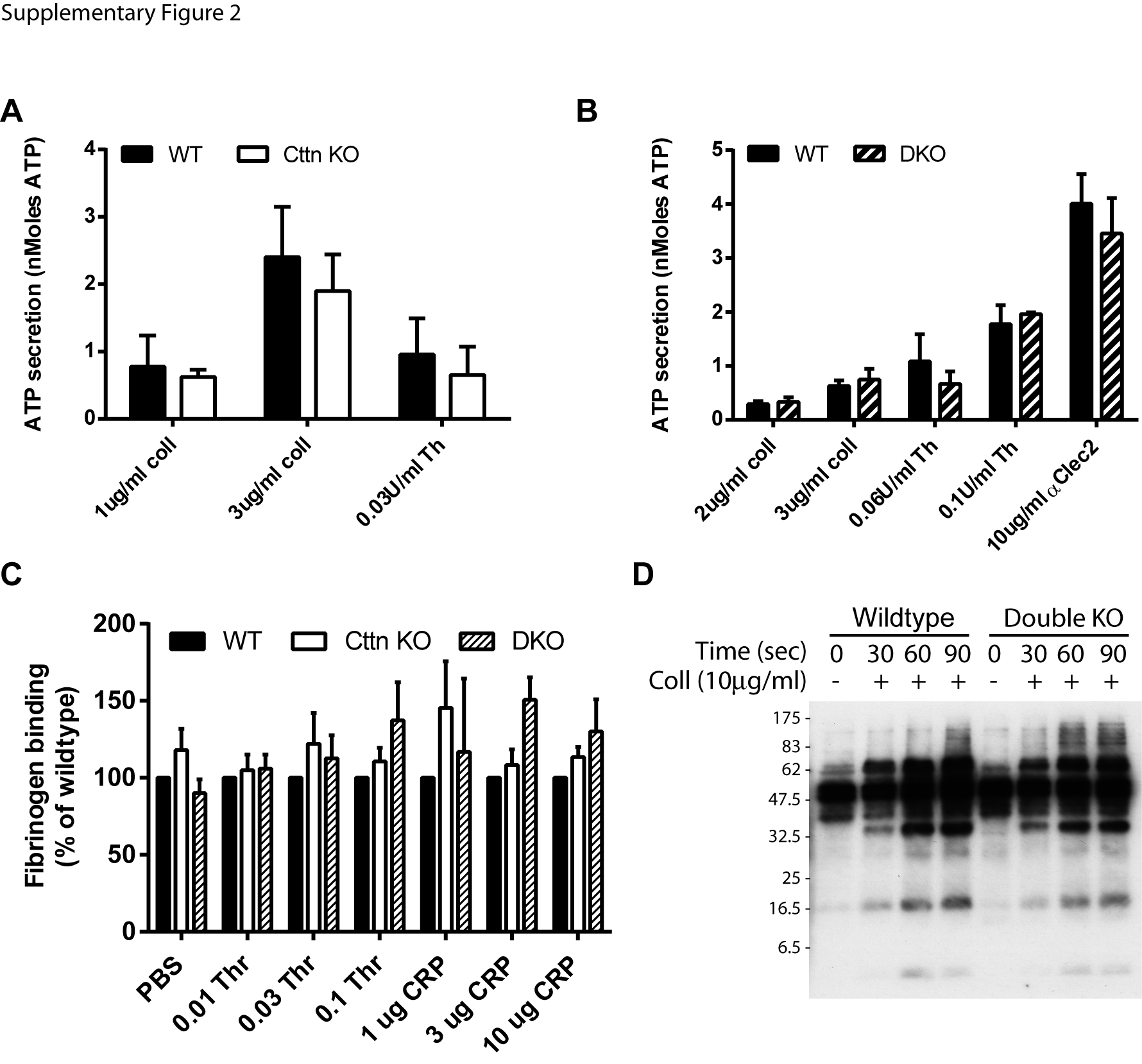

Supplement: Thomas et al Supplemental Figures [file iplt_a_1235688_sm0637.zip › SuppFig2.png]

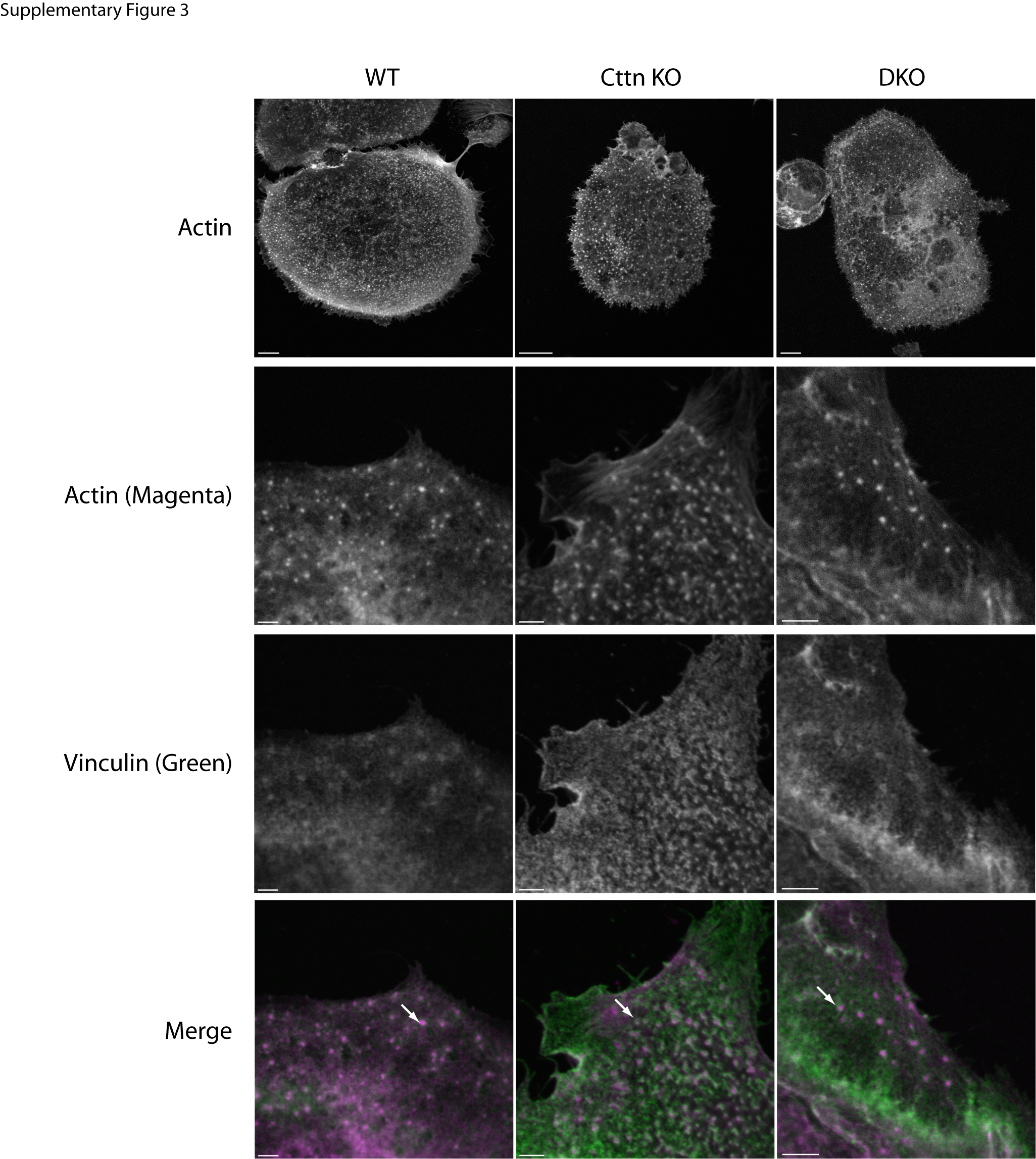

Supplement: Thomas et al Supplemental Figures [file iplt_a_1235688_sm0637.zip › SuppFig3.png]

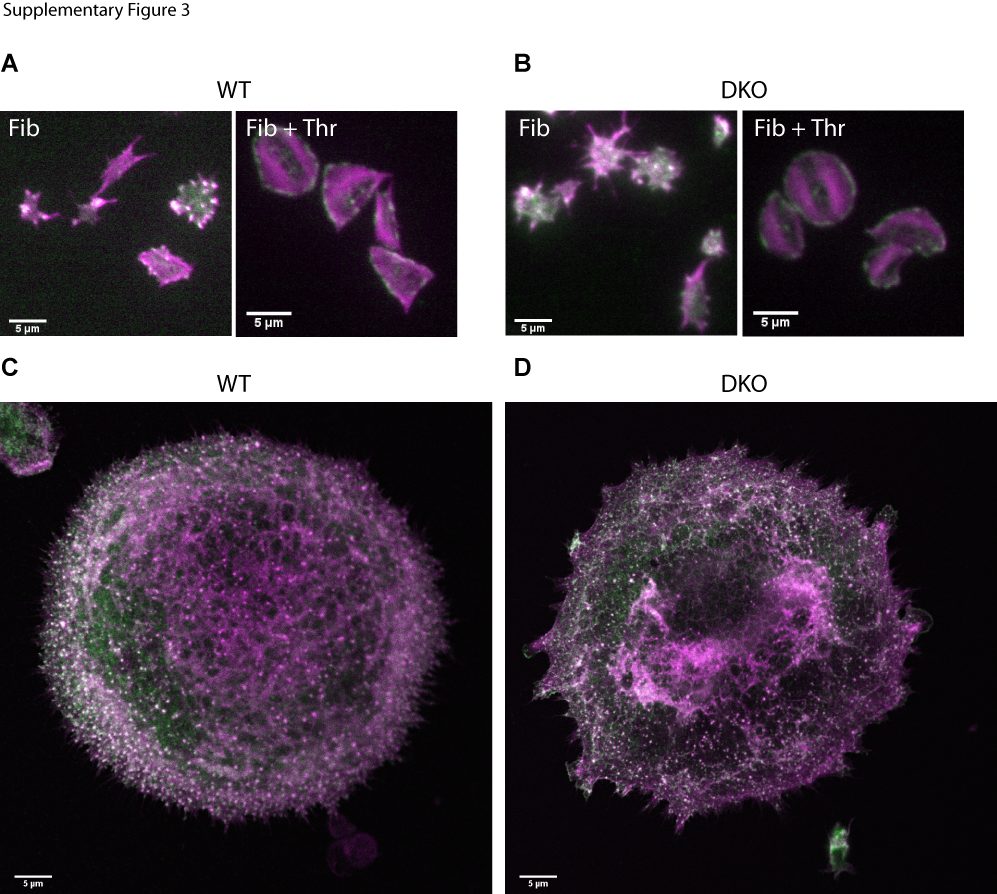

Supplement: Thomas et al Supplemental Figures [file iplt_a_1235688_sm0637.zip › SuppFig4.png]
